# Phenotypic and genetic classification of diabetes

**DOI:** 10.1007/s00125-022-05769-4

**Published:** 2022-08-12

**Authors:** Aaron J. Deutsch, Emma Ahlqvist, Miriam S. Udler

**Affiliations:** 1 Diabetes Unit and Center for Genomic Medicine, Massachusetts General Hospital, Boston, MA USA; 2 Program in Medical & Population Genetics, Broad Institute, Boston, MA USA; 3 Program in Metabolism, Broad Institute, Boston, MA USA; 4 Department of Medicine, Harvard Medical School, Boston, MA USA; 5 Genomics, Diabetes and Endocrinology, Department of Clinical Sciences in Malmö, Lund University Diabetes Centre, Lund University, Malmö, Sweden

**Keywords:** Cluster analysis, Disease subtypes, Genetics, MODY, Personalised medicine, Polygenic score, Precision medicine, Review, Type 1 diabetes, Type 2 diabetes

## Abstract

The historical subclassification of diabetes into predominantly types 1 and 2 is well appreciated to inadequately capture the heterogeneity seen in patient presentations, disease course, response to therapy and disease complications. This review summarises proposed data-driven approaches to further refine diabetes subtypes using clinical phenotypes and/or genetic information. We highlight the benefits as well as the limitations of these subclassification schemas, including practical barriers to their implementation that would need to be overcome before incorporation into clinical practice.

**Graphical abstract:**

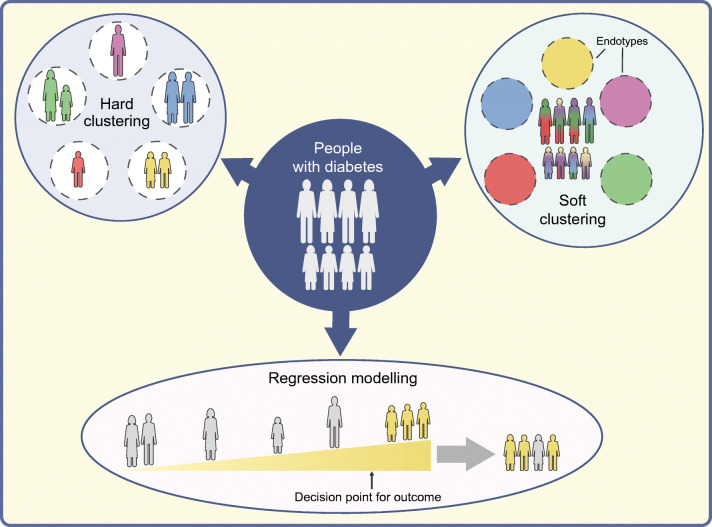

**Supplementary Information:**

The online version of this article (10.1007/s00125-022-05769-4) contains a slideset of the figures for download, which is available to authorised users..

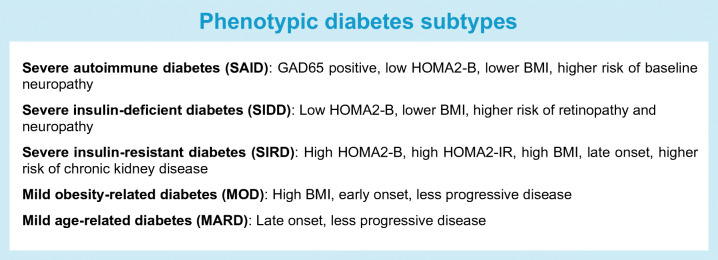

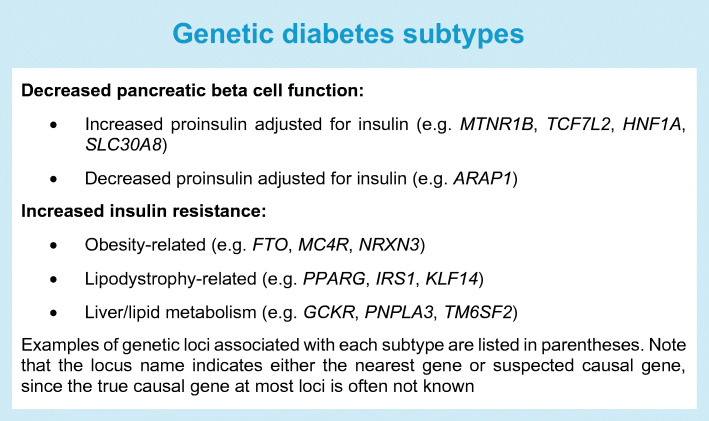





## Introduction

Diabetes is a heterogeneous group of diseases defined by chronically elevated blood glucose levels. This umbrella diagnosis is generally divided into several categories, including type 1 diabetes, type 2 diabetes, gestational diabetes and diabetes due to other causes (e.g. monogenic diabetes or medications), with the majority of diabetes cases attributed to type 2 (90–95%) or type 1 (5–10%) [1].

Various biomarkers can help establish the subtype of diabetes. For example, type 1 diabetes is strongly associated with the presence of islet autoantibodies, although these antibodies may be absent in up to 10% of individuals [2]. Additionally, monogenic diseases such as MODY can be diagnosed with a single genetic test. In contrast, no single biomarker can conclusively establish a diagnosis of type 2 diabetes, which is the default diagnosis of any individual with diabetes who does not fulfil the criteria for a more specific diabetes diagnosis [1]. In type 2 diabetes, hyperglycaemia can arise from different processes, ranging from insulin deficiency (in individuals with relative insulin sensitivity) to severe insulin resistance and hyperinsulinaemia; clinical presentations also vary greatly with respect to disease severity, risk of complications and response to therapy [3, 4].

Further subclassification of diabetes into more homogeneous groups offers the potential for improved, personalised treatment of diabetes [5]. Both phenotypic and genotypic information can provide more precise classifications, ideally elucidating distinct biological mechanisms that contribute to development of hyperglycaemia in a given person. Such patient stratification may allow a precision medicine approach to diabetes management, highlighting subsets of patients who are: (1) at highest risk for disease progression or particular complications; and/or (2) most likely to benefit from particular management strategies.

This review summarises the approaches that have been proposed for clinical phenotype-based and genetically based subclassifications of diabetes. Since the majority of subclassification approaches published to date have used either phenotypic or genetic data points as inputs and not both, we present these two approaches separately, although we note that this is an artificial delineation, as these approaches are complementary and may in theory converge on shared subtypes. Subclassification approaches also differ with regard to the starting patient population (e.g. all-comers with diabetes, those with type 2 diabetes, those who have experienced diabetic ketoacidosis), and thus the study populations are noted throughout.

We will first introduce phenotype-driven subtyping strategies, with a focus on those using algorithmic approaches, which have identified mostly subtypes of type 2 diabetes. Second, we will introduce genetic strategies for patient stratification where we discuss applications to monogenic diabetes, autoimmune diabetes and type 2 diabetes. Finally, we will review proposed strategies to stratify patients at risk for diabetes, as well as potential future directions for clinical implementation of the various subtyping approaches.

## Phenotype-driven subclassification strategies

**Fig. 1:**
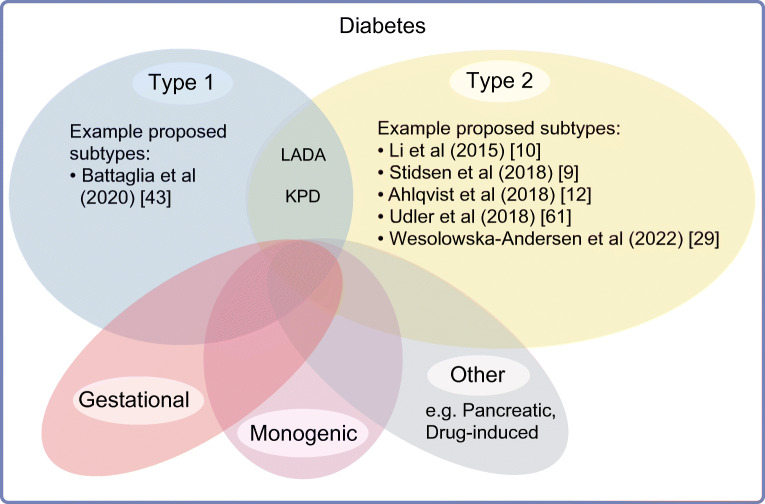
Diabetes subtypes. Diabetes has historically been classified as type 1, type 2, gestational or secondary to other causes (monogenic disease, pancreatic disease, drug-induced, etc.). Increasingly, there is recognition that overlap exists between these categories. Subtypes representing an overlap between type 1 and type 2 diabetes include LADA and ketosis-prone diabetes (KPD). Various strategies have been proposed to further divide type 1 and type 2 diabetes into subtypes, including the example publications listed. This figure is available as part of a downloadable slideset

Historically, the vast majority (>95% [1]) of individuals who develop diabetes outside of pregnancy have been placed into two subtypes that have been referred to as type 1 and type 2 since the 1950s, although they were recognised as distinct entities long before these terms were coined. These subtypes have been defined by clinical characteristics and have been updated over the years to incorporate new knowledge, such as the discovery of autoantibodies to pancreatic islet cells in type 1 diabetes in the 1970s [6, 7]. The non-discrete nature of these two categories has been well recognised, with conditions such as latent autoimmune diabetes in adults (LADA) and ketosis-prone diabetes representing individuals with clinical features overlapping with type 1 and type 2 diabetes (reviewed in more detail elsewhere e.g. [8]; Fig. 1).

Recognising the imprecision of the existing diagnostic categories of diabetes, various algorithms have been proposed to more objectively divide diabetes into subtypes based on phenotypic criteria, such as using blood-based estimates of insulin secretion capacity and insulin resistance [9], large-scale network analysis of phenotypes from electronic medical records [10], and presence or absence of autoantibodies and intact beta cell function in diabetes involving ketoacidosis (‘AB classification’) [11]. While all these approaches have supported the presence of heterogeneity within existing diabetes subtypes, they have either pertained to a small subset of all diabetes cases or have yet to be broadly replicated.

### Five clinical subtypes of diabetes at time of diagnosis

An algorithmic subclassification approach that has been arguably the most well replicated, including with findings of clinical consequences related to the subgroups, was proposed in 2018 by Leif Groop and colleagues (sometimes referred to as the Ahlqvist classification) [12]. Individuals with recently diagnosed diabetes from the All New Diabetics In Scania (ANDIS) study were grouped by phenotypic similarity based on six clinical variables selected to reflect important risk factors and aspects of the pathogenesis of diabetes: presence of GAD65 autoantibodies, age at diabetes diagnosis, BMI, HbA_1c_ at diagnosis, and homeostatic model assessment estimates of insulin secretion capacity (HOMA2-B) and insulin resistance (HOMA2-IR). Using these variables, the individuals with newly diagnosed diabetes were clustered using *k*-means and hierarchical clustering into five subtypes. The characteristics and stability of the clusters were replicated in three independent cohorts from Sweden and Finland as part of the initial publication.

The ANDIS clusters were named after their most defining trait: severe autoimmune diabetes (SAID) was defined by being GAD65 positive and thus included all individuals with type 1 diabetes and LADA. As expected, this group had low insulin secretion capacity, relatively low BMI and poor metabolic control (high HbA_1c_). Individuals in the severe insulin-deficient diabetes (SIDD) group were GAD65 negative but otherwise similar to SAID. SIDD had the highest risk of early diabetic retinopathy [12] and neuropathy [13]. Severe insulin-resistant diabetes (SIRD) was characterised by obesity, severe insulin resistance, high insulin secretion and late onset, but relatively low HbA_1c_. This group had a markedly higher risk of developing diabetic kidney complications, including chronic kidney disease (CKD), albuminuria and end-stage renal disease (ESRD). People with SIRD also had a higher prevalence of non-alcoholic fatty liver [12, 13]. The mild obesity-related diabetes (MOD) and mild age-related diabetes (MARD) subtypes were characterised by early onset (and obesity) and late onset, respectively.

### Replication of ANDIS subtypes in diverse populations

Since the first publication, replication of the ANDIS subtypes has been attempted in numerous cohorts of diverse populations, at times using different clustering methods and variables [13–19]. Overall, the five subtypes have been broadly reproducible, with a detailed summary of replication studies included elsewhere [20]. Several studies that used similar methods to the original study have closely replicated the characteristics of the five groups, including differences in risk of complications [13, 14, 18, 19, 21, 22]. While the same clusters were observed in several ethnicities, differences have been demonstrated both in proportions and in mean values of the variables used for classification [14, 23–26]. For example, a replication study in a Chinese cohort showed a larger proportion of SIDD individuals as well as generally lower BMI and earlier diabetes onset [21]. Studies using alternative clustering variables or methods have shown partially consistent results [15–17]. For example, a study in a large Indian cohort of individuals with diabetes identified an additional cluster of individuals with both insulin deficiency and insulin resistance [16]. In a study by the Risk Assessment and Progression of Diabetes (RHAPSODY) consortium, addition of HDL as a cluster variable divided the MARD cluster into two subgroups [17]. It is often difficult to discern if differences in clustering results, subtype proportions and characteristics are true population differences or study-specific due to methodology or patient inclusion; caution should be applied in interpreting studies until replicated.

While the classification was developed in populations with recently diagnosed diabetes, the clusters have also been studied in populations with diabetes of longer duration (e.g. the Finnish Diabetes Register in Vasa [DIREVA] cohort subset in [12]) and in populations with longitudinal follow-up, using repeated measures from the same individuals to assess whether movement between clusters occurs over time [13, 15]. One such study followed 367 individuals from the German Diabetes Study (GDS) over a period of 5 years after diabetes diagnosis [13]. The proportion of individuals allocated to the same cluster at baseline and 5 year follow-up was on average 77% but varied by cluster (20% SIDD, 82% SAID, 51% SIRD, 79% MOD and 82% MARD), suggesting some movement, particularly for individuals in the SIDD cluster [13]. Potential explanations for cluster reassignment include exclusion criteria in GDS, such as exclusion of individuals with poor glycaemic control (HbA_1c_ >74.9 mmol/mol [9%]) leading to fewer true SIDD cases [27]; resolution of beta cell stress after treatment of initial severe hyperglycaemia; or disease progression, such as development of insulin resistance over time. While HbA_1c_ in treated SIDD cases remained high at the time of subsequent cluster assignment, the relative difference in HbA_1c_ values compared with SIRD, MOD and MARD was not as marked, impeding cluster assignment [13]. Ongoing follow-up studies in DIREVA and ANDIS will provide more information about the progression between clusters over time.

### Genetic understanding of ANDIS subtypes

Recently, the original clusters from the ANDIS cohort were characterised genetically using genome-wide association and polygenic score analysis [28]. Polygenic scores were constructed to capture the aggregate effect of multiple variants affecting a trait of interest across the genome. The authors used scores composed of variants associated with type 2 diabetes weighted by their genetic effect on measures of insulin secretion and sensitivity. The SIRD subtype stood out as not associated with any polygenic score reflecting insulin secretion (i.e. insulin secretion rate or corrected insulin response during glucose tolerance test). Additionally, only the SIRD subtype was significantly associated with the polygenic score for fasting insulin. Polygenic scores for BMI (including variants reaching genome-wide association with BMI) were most strongly associated with the MOD and SIRD subtypes but not with MARD. Polygenic scores for type 1 diabetes were specific to the SAID subtype with no overrepresentation in SIDD compared with the other GAD65-negative subtypes or diabetes-free control groups, arguing against a substantial role of autoimmunity in the relative insulin deficiency seen in most individuals with the SIDD subtype. A SNP in the *LRMDA* locus was also found to be uniquely associated with the MOD subtype [28]. These results showed that there are aetiological differences between the subtypes and that subtype-specific loci can be identified; future studies with larger sample sizes are likely to show more subtype-specific associations.

### Subclassification of diabetes without distinct subgroups

**Fig. 2:**
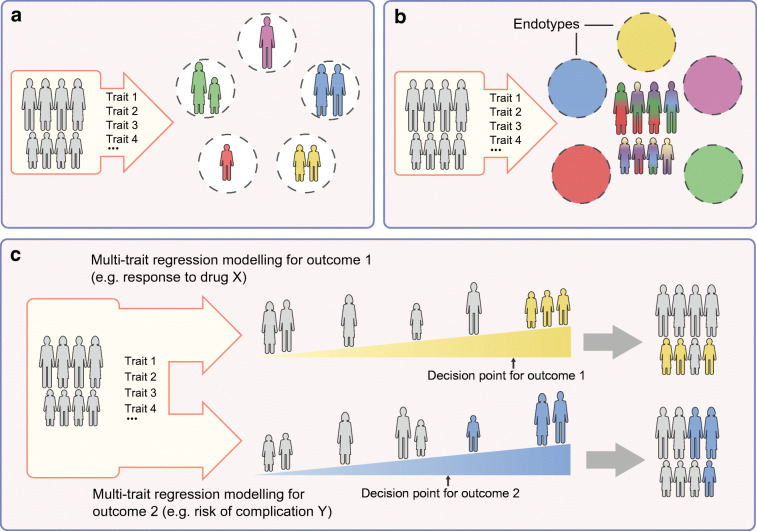
Strategies for identifying diabetes subtypes. (**a**) Hierarchical (‘hard’) clustering distributes people into discrete subtypes. These clusters are defined using a series of traits, which may include phenotypic and/or genotypic criteria. (**b**) In a ‘soft’ clustering approach, discrete subtypes are also defined using a series of traits; however, people may have features belonging to more than one cluster. Clusters that represent a distinct pathobiological mechanism may be referred to as endotypes. (**c**) Alternatively, clinical traits may be integrated into a regression model, yielding a continuous measurement of various outcomes (e.g. response to a certain drug or risk of developing a certain complication). Clinical decisions (e.g. to start a certain medication) are implemented for people who fall above a specified threshold. This figure is available as part of a downloadable slideset

While there is clinical appeal to a hierarchical (‘hard’) clustering strategy, in which individuals are assigned to a single cluster or diabetes subtype, ‘soft’ clustering approaches allow individuals with diabetes to have contributions from multiple subtypes (Fig. 2a,b). This approach was recently taken in 726 individuals with type 2 diabetes in the Innovative Medicines Initiative (IMI) Diabetes Research on Patient Stratification (DIRECT) study, where a novel clustering approach that considered 32 anthropometric, clinical and biochemical phenotypes identified four quantitative profiles [29]. Most individuals had intermediate characteristics related to more than one of the four profiles; however, 101 individuals (~14%) had extreme phenotypes of a single profile and were considered ‘archetypes’. The four archetypes differed in glycaemic progression and omics signals, but have not yet been replicated in an independent dataset [29].

An alternative strategy to stratify patients is to use continuous variables integrated into a regression model, yielding a continuous measurement of various outcomes (e.g. risk of developing a certain complication or glucose-lowering effect of a particular medication; Fig. 2c). Thresholds can be chosen to implement clinical decisions, such as starting a given medication. Under this approach, clinical endpoints can be modelled for an individual patient using all relevant patient information, rather than considering the collective risk of the endpoint in those assigned to a given cluster. Dennis and colleagues have used this approach to model glycaemic progression (HbA_1c_ change over time), incidence of kidney disease and glycaemic response to medications in a reanalysis of two clinical trials [18, 30]. The results were comparable or at times superior to the ANDIS cluster-based approach; for example, eGFR at baseline was a better predictor of time to CKD (defined as eGFR below 60 ml min^−1^ 1.73 m^−2^) than cluster membership, and modelling of simple clinical features (sex, BMI, age at diagnosis, baseline HbA_1c_) outperformed the clusters for treatment selection, as measured by HbA_1c_ lowering in SIRD [18]. When considering these results, it is important to recognise that the cohorts were selected to exclude the most severe cases (that would mostly belong to SIDD and SIRD), which could lead to an underestimation of differences between clusters. In accordance, this study found no significant difference in risk of complications after adjustment for baseline eGFR, whereas studies of the ANDIS clusters found significant differences, with larger effect sizes for more severe kidney disease and with longer duration of diabetes [12, 31]. For treatment selection, the continuous models only evaluated HbA_1c_ as the endpoint, whereas change in insulin resistance and risk of kidney disease would be as important to evaluate benefit in the SIRD cluster, which has relatively good metabolic control. Of course, an important limitation of HbA_1c_ lowering as a single endpoint is that it may be influenced by hypoglycaemic adverse events. In spite of the limitations, modelling using continuous traits is a valuable approach that can provide improved prediction for specific complications, and the two strategies can be regarded as complementary [32].

### Clinical translation of phenotypic subclassification

Before novel subclassification approaches are implemented into clinical practice, there first needs to be robust evidence of benefit to patients. While multiple studies have supported the value of ANDIS cluster membership for prediction of diabetes complications (e.g. [13]), there are limited data on response to treatment. As mentioned above, reanalysis of A Diabetes Outcome Prevention Trial (ADOPT) and Rosiglitazone Evaluated for Cardiovascular Outcomes and Regulation of Glycemia in Diabetes (RECORD) indicated benefit of particular drugs for certain clusters, such as sulfonylureas for MARD and thiazolidinedione for SIRD [18]. In the Outcome Reduction with Initial Glargine Intervention (ORIGIN) trial, the subtype with the greatest glycaemic response to glargine, a long-acting insulin, compared with standard care was the SIDD subtype, where it decreased occurrence of hyperglycaemia (defined as a mean post-randomisation HbA_1c_ ≥47.5 mmol/mol [6.5%]) by 13% in comparison with the MARD subtype [22]. While promising, further studies are needed before clinical recommendations can be made based on the subclassification.

For a phenotypic subtyping approach that involves clustering or regression modelling to be applied in clinical practice, there would need to be: (1) measurement of the defining phenotypes in a given patient; and (2) real-time analysis using the patient’s phenotypes to determine subtype or outcome risk. While measurement of complex phenotypes, such as HOMA estimates, may not be widely available, studies have demonstrated that simple measurements sometimes can be used as surrogates for complex phenotypes [15, 17, 33]. However, such simpler surrogates may not always be adequate, as for example no study has enabled identification of the SIRD cluster without some measure of C-peptide or insulin. For real-time mapping of the patient’s traits to clusters or regression model outcomes, there are efforts underway to develop decision support tools, although notably a challenge that emerges is ability to map a given patient’s clinical data to an appropriately representative cohort (e.g. with similar ethnicity) for determination of that patient’s cluster membership or modelled outcome risk.

## Genetic subtyping approaches

In addition to phenotypic information, genetic information can be used to subclassify diabetes. The clearest example of a genetic subtype is seen with monogenic diabetes, where a diabetes subtype is defined by a single blood test. Establishing a diagnosis of monogenic diabetes has important clinical implications, informing timing and severity of disease onset; response to therapy; and expected disease progression and complication risk. Beyond monogenic diabetes, genetic approaches involving polygenic scores have aimed to: (1) improve delineation of diabetes subtypes; and (2) improve subclassification within type 1 and type 2 diabetes.

### Monogenic diabetes

Arguably, the most objectively defined subcategories of diabetes are monogenic subtypes, whereby the majority of diabetes risk comes from variation in a single gene. Monogenic diabetes accounts for approximately 0.4% of all diabetes [34] and 1–6% of paediatric diabetes cases [35]. Of course, individuals with monogenic diabetes were initially described based on phenotypic features before causal genes were discovered and genetic testing became more widely available; however, genetic testing can now provide definitive diagnoses for patients and has allowed better characterisation of distinct genetic disease subtypes. For example, 80% of individuals with monogenic diabetes have been estimated to be misdiagnosed as having type 1 or type 2 diabetes [36], and thus correctly identifying monogenic diabetes is essential for refining the heterogeneity of diabetes.

The most common form of monogenic diabetes, MODY, is usually inherited in an autosomal dominant fashion and is characterised by pancreatic beta cell dysfunction [35]. MODY is classically recognised as: (1) diabetes onset before age 35; (2) with strong family history of similar diabetes; and (3) lack of characteristics of type 1 diabetes (e.g. no islet autoantibodies) and of type 2 diabetes (e.g. no marked obesity) [35]. Increasingly, there is also appreciation that not all individuals with MODY meet all criteria [37] and that common genetic variation contributes to clinical features of monogenic disease [38], such as age of diagnosis [39]. Nevertheless, knowledge of the genetic subtype of diabetes has very important implications for a patient’s clinical course and response to treatment. For example, individuals with MODY caused by mutations in *GCK* (*GCK*-MODY) have mild, non-progressive hyperglycaemia present from birth with low risk of complications and typically do not require treatment [40]; individuals with *HFN1A-* and *HNF4A-*MODY may achieve excellent control with sulfonylureas [41] or glucagon-like peptide 1 (GLP-1) receptor agonists [42], removing the need for insulin.

### Diabetes classification informed by type 1 diabetes genetics

Autoimmune diabetes is increasingly recognised as involving more than classic type 1 diabetes; it represents a heterogeneous group of diseases with differences in age of onset, rates of progression and rates of complications (described in [43]). Genetic approaches have been used both to help distinguish other forms of diabetes from type 1 diabetes, as well as to evaluate evidence of distinct subtypes within autoimmune diabetes.

Genetic contribution to type 1 diabetes, as quantified in a polygenic score, has demonstrated exciting clinical potential to improve delineation of diabetes subtypes. With growing discovery of genetic loci associated with type 1 diabetes and increased ability to model the HLA region’s impact on disease risk, the type 1 diabetes polygenic score has evolved over the years. One of the most recent versions, a polygenic score constructed from 67 genetic variants, can predict the risk of developing type 1 diabetes in children with an AUC of the receiver operating characteristic (ROC) curve of 0.92 [44], where a value of 1.0 indicates a perfectly accurate test. Type 1 polygenic scores have also demonstrated value in addressing diagnostic uncertainty, such as discrimination of type 1 diabetes from type 2 diabetes [44, 45], MODY [46] and syndromic autoimmune monogenic diabetes [47].

Genetic studies have also evaluated a potential subtype of autoimmune diabetes, LADA. The LADA diagnosis is considered to apply to a subgroup of individuals with autoantibodies who initially present with diabetes similar to type 2 diabetes, but subsequently require insulin [48, 49]. LADA has been defined using various criteria, and a question has been raised as to whether it exists as a distinct clinical entity or represents a heterogeneous mix of people with type 1 and type 2 diabetes (who by chance have background levels of autoantibody positivity) [50]. The largest genome-wide association study (GWAS) for LADA found that most loci were associated with type 1 diabetes (e.g. *HLA*, *INS*, *PTPN22*), although some genes such as *TCF7L2* were shared by LADA and type 2 diabetes [51]. An analysis of polygenic scores in 978 LADA cases demonstrated that a type 1 diabetes polygenic score was more predictive of LADA than a type 2 diabetes polygenic score (AUC of ROC curve 0.67 vs 0.57), although neither score offered substantial discriminatory power [52]. While such findings suggest that LADA has genetic contribution from both type 1 and type 2 diabetes, they do not exclude the possibility that LADA comprises a heterogeneous mix of people with type 1 and type 2 diabetes rather than being a distinct diabetes subtype. Notably, the latter study also assessed the distribution of the type 1 diabetes polygenic score (composed of 69 SNPs) in people with type 1 diabetes compared with people with LADA who were positive for both GAD and islet antigen 2 (IA-2) antibodies and found that the mean score value was significantly lower in the LADA group [52]. This finding suggests that LADA may be genetically distinct from type 1 diabetes, and it is unlikely that the LADA cases represented mixing of individuals with type 1 and type 2 diabetes, given the low likelihood that someone with type 2 diabetes would have two autoantibodies elevated just by chance. It is certainly possible that type 1 diabetes genetic risk exists on a spectrum, and people with moderate genetic risk (perhaps encompassing LADA) may develop a milder phenotype. Additionally, while the type 1 diabetes polygenic score applied in [52] could not definitively identify individuals with LADA, the score may help identify a subset of patients who are more likely to require insulin; among patients with type 2 diabetes and GAD65 autoantibodies, 48% of patients with a (30 variant [45]) type 1 diabetes polygenic score above the 50th percentile required insulin within 5 years of diabetes diagnosis, compared with 18% of patients with scores below the 5th percentile [53].

### Diabetes classification informed by type 2 diabetes genetics

Similar to type 1 diabetes, polygenic scores can identify individuals at risk of developing type 2 diabetes; however, recently developed scores for type 2 diabetes involving thousands of genetic variants (‘global extended polygenic scores,’ described in [54]) only reach AUCs of the ROC curve of 0.73 in discriminating people with type 2 diabetes from control groups after adjustment for age and sex [54]. Focusing on individuals with the top 5% of type 2 diabetes polygenic scores can identify people with a 4.5-fold increased risk of type 2 diabetes compared with the rest of the population [54, 55]. Compared with type 1 diabetes polygenic scores, the type 2 diabetes polygenic score provides less ability to distinguish between type 1 and type 2 diabetes (AUC of ROC curve 0.64 [95% CI 0.63, 0.66]) [45]. Thus, current versions of the type 2 diabetes polygenic score have limited ability to definitively diagnose type 2 diabetes or delineate it from other diabetes subtypes.

A separate line of research has focused on whether genetic information can be used to help identify genetic subtypes within type 2 diabetes, represented as disease driven by particular genetic pathways. There has been tremendous discovery of genetic variants associated with type 2 diabetes, with well over 500 loci identified to date [56]. Such findings have great potential to inform disease biology and improve understanding of why patients develop disease. Clinical translation of these findings from genetic association studies has been limited, however, in large part because the majority of genetic signals fall within non-protein-coding regions of the genome, making it challenging to pinpoint causal variants and genes [57]. As a result, identifying genetic pathways predisposing to type 2 diabetes is not trivial. Early efforts to systemically connect type 2 diabetes loci to pathways initially focused on associations of loci with glycaemic traits [58–60] and broadly connected loci to ‘hard’ clusters related to beta cell function and insulin action (Fig. 2a). Notably, in these studies the majority of the loci were grouped in a single ‘unclassified’ cluster, even though several had known biological mechanisms (e.g. *HNF1A*, *KCNJ11*).

In 2018, two studies employed an alternative approach clustering variants and multiple glycaemic and non-glycaemic traits using ‘soft clustering’ (Fig. 2b), intended to better capture the pleiotropic nature of variants involved in more than one genetic pathway [57, 61]. These two studies generated a set of five broadly overlapping genetic clusters that were more readily interpretable than prior efforts. Each cluster related to a disease mechanism that could be inferred from the set of top-weighted genetic loci and associated clinical traits: two clusters related to decreased beta cell function (e.g. *MTNR1B*, *TCF7L2*, *HNF1A*, *SLC30A8*, reduced disposition index, increased proinsulin adjusted for insulin levels; and *ARAP1/STARD10*, reduced proinsulin adjusted for insulin levels), and three related to mechanisms of insulin resistance, mediated through: (1) obesity (e.g. *FTO*, *MC4R*, *NRXN3*, increased percentage body fat, BMI); (2) abnormal fat distribution or ‘lipodystrophy’ (e.g. *PPARG*, *IRS1*, *KLF14*, increased fasting insulin, triglycerides, reduced BMI); and (3) impaired liver/lipid metabolism (e.g. *GCKR*, *PNPLA3*, *TM6SF2*, reduced triglycerides). The effort by Mahajan and colleagues also identified a sixth cluster with mixed phenotypic features [57].

In theory, genetic clusters of loci can identify subsets of individuals for whom type 2 diabetes risk is primarily driven by a specific pathway, indicating a genetic subtype of disease. A person’s genetic risk for a given cluster can be calculated using a cluster-specific partitioned polygenic score that generates a weighted sum of the number of variants carried for a given cluster, with the weights corresponding to the strength of each variant’s membership to that cluster [54]. Using the five genetic clusters described in [61] (sometimes referred to as the Udler clusters), individuals with type 2 diabetes who fell in the top 10th percentile of just one cluster-specific polygenic score were shown to have distinct clinical features [61]. For example, those with a high burden of genetic variants related to beta cell dysfunction had significantly lower C-peptide levels compared with all others with type 2 diabetes, indicating that their diabetes represented relative insulin deficiency [61]. Other key defining features of the genetic subtypes included elevated BMI in the ‘obesity’ genetic subtype, elevated C-peptide and reduced HDL-cholesterol in the ‘lipodystrophy’ genetic subtype and reduced triglyceride levels in the ‘liver/lipid’ subtype. Additionally, these type 2 diabetes partitioned polygenic scores have been shown to be associated with comorbid metabolic diseases. For instance, hypertension was more likely in people with a higher score in the ‘obesity’ cluster or the ‘lipodystrophy’ cluster; people with a higher ‘liver/lipid’ polygenic score were more likely to have CKD but less likely to have coronary artery disease [61, 62].

Recent work investigating adipose mesenchymal-derived stem cells has demonstrated, using lipocyte cell painting, that the ‘lipodystrophy’ cluster polygenic score identifies a distinct cellular-level phenotype. Study participants with the top 20% lipodystrophy polygenic score values had obvious differences in cellular features, with increased mitochondrial activity and decreased lipid accumulation, compared with those with the bottom 20% scores [63]. Remarkably, this cellular profile was also shared with single-gene perturbations for monogenic lipodystrophy genes, supporting convergence of polygenic and monogenic diabetes pathways [63].

### Clinical translation of genetic subclassification

Genetic testing for monogenic diabetes is part of current clinical practice, although barriers to its use include recognition of potential cases appropriate for testing and access to testing. The polygenic diabetes scores are not currently part of general clinical practice. Given the high discriminatory ability of the type 1 diabetes score, there is a potential role for it in improving diabetes classification in practice, for example, by applying it in patients with diagnostic uncertainty. In contrast, both the full and cluster-specific polygenic scores for type 2 diabetes currently have insufficient predictive ability to warrant use in standard practice [38, 62]. While all five subgroups in the genetically driven subclassification of type 2 diabetes described in [61] had distinct replicable clinical phenotypes, phenotypic differences between subgroups were quantitatively small and unlikely to be appreciated clinically on an individual patient level [62]. Nevertheless, polygenic scores related to type 2 diabetes have the potential to become more predictive in future iterations, particularly with the inclusion of rarer genetic variation [64], which may lead to future use in clinical practice.

Identification of more precise genetic subtypes of type 2 diabetes may enable targeted therapies; for example, patients with high genetic risk for beta cell dysfunction may benefit from early initiation of insulin, whereas patients with high genetic risk for obesity-related diabetes may benefit from insulin-sensitising agents. Such hypotheses need to be tested and will require access to large clinical trials with genetic data.

A significant challenge for clinical translation of polygenic subtypes of type 2 diabetes is that the majority of large-scale genetic studies have been conducted in individuals of European ancestry, although there are increasing efforts for datasets to present more diverse ancestral groups [56, 65]. Further investigation is needed to include more diverse populations, particularly to avoid exacerbation of health disparities [66].

## Subclassification of people at risk for type 2 diabetes

Clustering methods have also been performed in individuals who do not have type 2 diabetes but are at elevated risk of developing the disease. Wagner and colleagues used a hybrid approach that combined phenotypic values (measures derived from oral glucose challenge and MRI-measured body fat distribution and liver fat content) and polygenic risk of type 2 diabetes, identifying six clusters with different propensities to develop diabetes and diabetes-related complications [33]. Interestingly, some of the clusters had clinical features similar to the ANDIS clusters; yet, despite the similar features, the people in the at-risk diabetes clusters who developed diabetes were not consistently members in the corresponding ANDIS cluster. Nevertheless, studying people at risk for type 2 diabetes illustrates how improved stratification would be valuable for more targeted efforts to prevent diabetes and related complications.

The efforts aimed at identifying genetic subtypes of type 2 diabetes can also potentially be applied to stratify people at risk of type 2 diabetes before they develop symptomatic disease. For instance, in an analysis of large cohorts containing people with and without diabetes, people with an elevated ‘lipodystrophy’ cluster polygenic score had an increased risk of hypertension even after adjusting for type 2 diabetes status [62], suggesting that pathway-specific polygenic scores can predict risk of specific combinations of future diseases (e.g. type 2 diabetes and hypertension).

## Future directions

Phenotypic and genotypic patient characteristics offer complementary approaches to classify diabetes subtypes. Further research is therefore also needed to determine how best to integrate both types of data together to improve disease subclassification. In addition, it is possible that deeper phenotyping of patients, including a broader set of phenotypic traits, may more precisely distinguish between various subtypes of diabetes (e.g. analysing multiple islet cell autoantibodies, not just GAD65, in the ANDIS classification).

Although type 2 diabetes primarily affects adults, it is increasingly being diagnosed in children, making it more challenging to discern known subtypes in this age group [67]. Additionally, the subclassification approaches described in this review have almost exclusively been replicated in adult populations, not paediatric. Further work is needed to apply and refine subclassification approaches for paediatric diabetes.

Once subtypes of diabetes have been identified and replicated, an important next step is demonstration of clinical significance. Clinical trials offer an important such opportunity, particularly when cardiovascular outcomes are measured. Limited analyses of clinical trials using phenotypic stratification approaches have been performed, as described, with identification of both subtypes and clinical features associated with response to particular medications [18, 22]. Fewer such analyses involving genetic subtypes have been performed to date (e.g. [68]). Wider access to clinical trial data will be critical for hypothesis testing and validation with replication that any diabetes subclassification has clinical utility. Additionally, clinical translation for subtyping approaches that involve algorithmic modelling will require decision support tools to facilitate integration of available information into clinical care, as well as continued inclusion of diverse populations to ensure broad and equitable translation of findings.

In conclusion, the existing subclassification of diabetes into predominantly type 1 and type 2 is increasingly recognised as insufficient to capture the heterogeneity of patient presentations, disease course, response to medications and risk of complications. Emerging subclassification schemas with more refined subgroups, involving phenotypic and genetic data, have already demonstrated reproducibility and in some instances evidence of clinical utility. Further study of the existing approaches, as well as novel integrated methods to redefine diabetes subtypes, will be necessary to determine when and how best to bring these approaches into mainstream clinical practice.

## Supplementary Information

Below is the link to the electronic supplementary material.

Supplementary file1 (PPTX 293 kb)

## Abbreviations

ANDIS: All New Diabetics In Scania
CKD: Chronic kidney disease
DIREVA: Diabetes Register in Vasa
GDS: German Diabetes Study
LADA: Latent autoimmune diabetes in adults
MARD: Mild age-related diabetes
MOD: Mild obesity-related diabetes
ROC: Receiver operating characteristic
SAID: Severe autoimmune diabetes
SIDD: Severe insulin-deficient diabetes
SIRD: Severe insulin-resistant diabetes
