# Supplementary material for: Phenotypic and genetic classification of diabetes
Source: Diabetologia. 2022 Aug 12;65(11):1758–69. doi: 10.1007/s00125-022-05769-4 (PMC9522707; doi:10.1007/s00125-022-05769-4)
Supplement: Supplementary file 1 — Supplementary file1 (PPTX 293 kb) [file 125_2022_5769_MOESM1_ESM.pptx]

## Slide 1
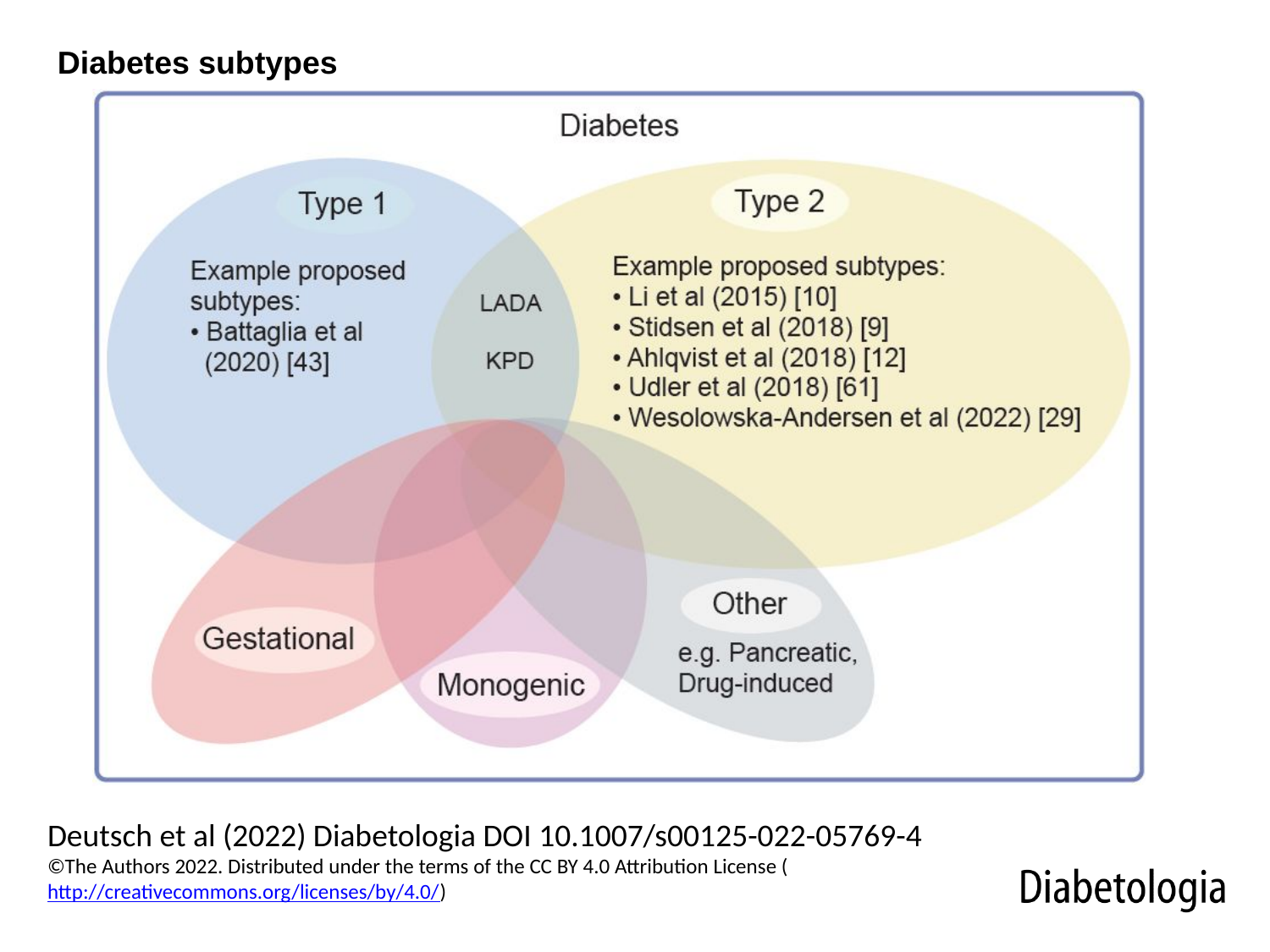

Diabetes subtypes
Deutsch et al (2022) Diabetologia DOI 10.1007/s00125-022-05769-4
©The Authors 2022. Distributed under the terms of the CC BY 4.0 Attribution License (http://creativecommons.org/licenses/by/4.0/)

## Slide 2
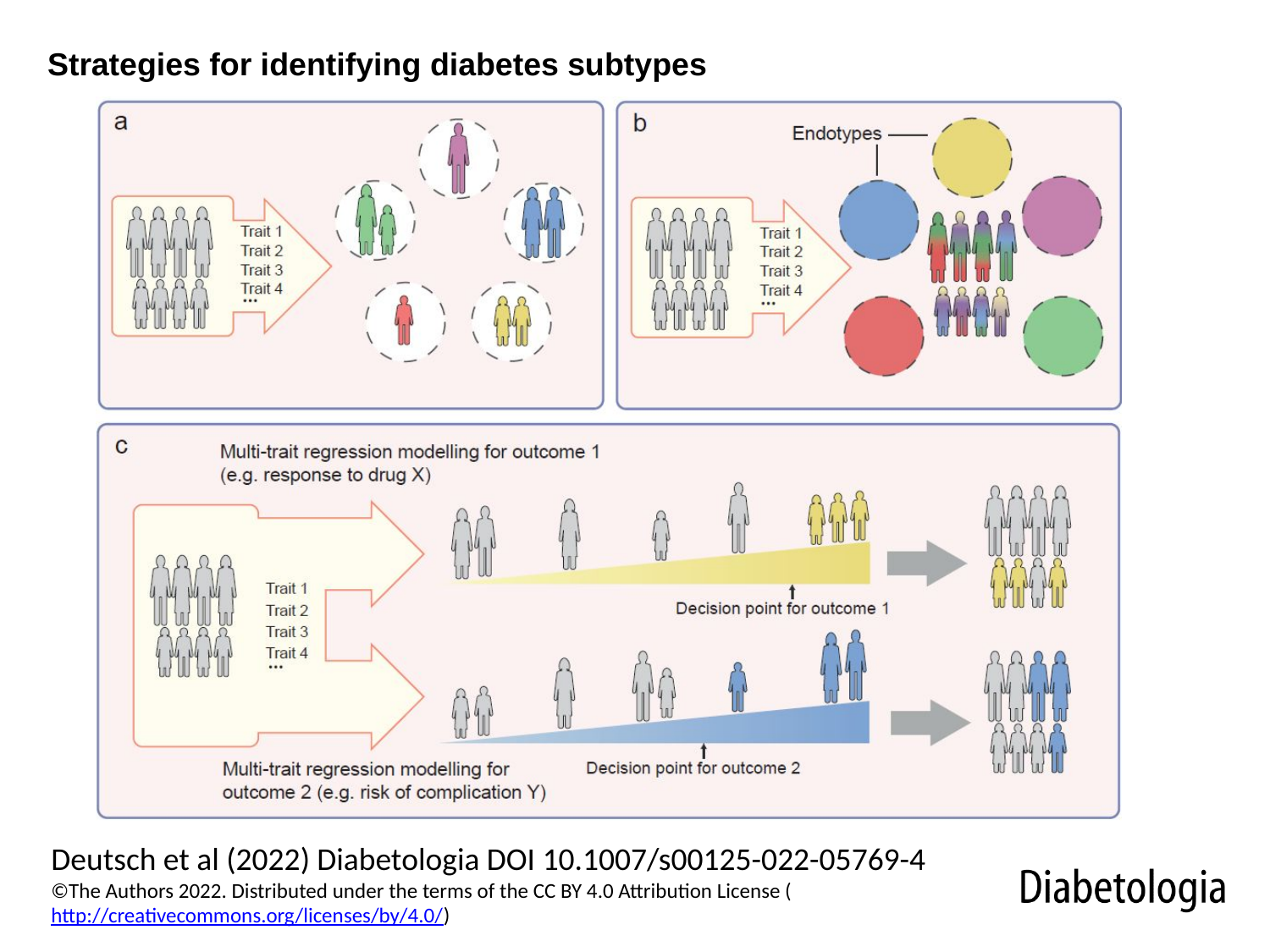

Strategies for identifying diabetes subtypes
Deutsch et al (2022) Diabetologia DOI 10.1007/s00125-022-05769-4
©The Authors 2022. Distributed under the terms of the CC BY 4.0 Attribution License (http://creativecommons.org/licenses/by/4.0/)
